# Multiple origins and functions: evolutionary pathways of HSP70 proteins in viruses

**DOI:** 10.1099/jgv.0.002242

**Published:** 2026-03-10

**Authors:** Ayoub Maachi, Santiago F. Elena

**Affiliations:** 1 Regional Center for Agronomic Research of Agadir, National Institute for Agronomic Research (INRA), Inezgane, 86350, Agadir, Morocco; 2 Instituto de Biología Integrativa de Sistemas, CSIC-Universitat de València, Paterna, 46980 Valencia, Spain; 3 Santa Fe Institute, Santa Fe, NM 87501, USA

**Keywords:** gene transfer, heat shock protein 70 superfamily, viruses

## Abstract

Heat shock protein 70s (HSP70s) are highly conserved molecular chaperones found across all domains of life, where they play essential roles in cellular stress responses. Whilst HSP70 homologues have been previously identified in closteroviruses that have ssRNA genomes, their broader presence and evolutionary history in viruses remain poorly understood. In this study, we conducted a comprehensive search of viral protein databases and identified HSP70 homologues in viruses beyond those with ssRNA genomes, including examples with dsDNA genomes in the class *Megaviricete*. These viral HSP70s exhibit diverse gene organizations, copy numbers and structural features. Notably, HSP70s of viruses from *Megaviricetes* showed up to three gene copies per genome and distinct structural motifs, whilst those from closteroviruses displayed higher sequence and structural diversity, suggesting faster evolutionary rates. Structural and phylogenetic analyses revealed two major clusters of viral HSP70s, with dsDNA virus HSP70s closely resembling those of their protist hosts, supporting the hypothesis of horizontal gene transfer. In contrast, ssRNA virus HSP70s formed a distinct, highly divergent group. Our findings suggest multiple independent acquisitions of HSP70 genes by viruses and provide new insights into their evolutionary trajectories and potential functional adaptations.

## Introduction

Heat shock proteins (HSPs) are a highly conserved family of molecular chaperones found across all domains of life, including bacteria, archaea and eukaryotes [1]. These proteins are essential for maintaining cellular proteostasis, particularly under conditions of physiological stress such as heat, oxidative damage or infection. Among the various HSP families, the 70 kDa HSPs (HSP70s) are among the best studied due to their central role in protein folding, assembly, translocation and degradation [2]. Structurally, HSP70s are composed of a nucleotide-binding domain (NBD) and a substrate-binding domain (SBD), connected by a flexible linker. The SBD itself includes a substrate-binding pocket and a lid domain, often ending with a conserved EEVD motif that mediates interactions with co-chaperones [3,4]. Large HSPs such as HSP110 and Grp170, which are homologous to HSP70s, also contribute to protein quality control and are considered part of the HSP70 superfamily [5,7].

In the context of viral infections, HSP70s play dual roles. On the one hand, they can inhibit viral replication by interfering with viral protein function or stability [8]. On the other hand, viruses can exploit host HSP70s to facilitate their own entry, replication and assembly. For example, HSP70s have been shown to act as viral receptors [9], assist in membrane translocation [10], stabilize viral ribonucleoproteins [11] and support virion assembly [12]. Despite their functional importance during infection, relatively little is known about whether viruses themselves encode HSP70 homologues. To date, the best-known examples are found in the plant-infecting closteroviruses that have ssRNA genomes, which encode HSP70-like proteins believed to have been acquired from host mRNAs via heterologous recombination [13,14]. These viral HSP70s retain conserved ATPase domains but show divergence in their C-terminal regions, and they function in viral movement between plant cells [15].

In this study, we aimed to systematically investigate the presence, diversity and evolutionary origins of HSP70 proteins encoded by viruses. We conducted a comprehensive search of viral protein sequences in the NCBI database, identifying HSP70 homologues in viruses that have both ssRNA and dsDNA genomes, including members of the *Alsuviricetes* and *Megaviricetes* classes. We analysed their gene organization, structural features, sequence diversity, phylogenetic relationships and possible functional diversification. Our findings reveal multiple independent acquisitions of HSP70 genes by viruses, distinct evolutionary trajectories between ssRNA and dsDNA viruses and potential horizontal gene transfer (HGT) events from host organisms, particularly protists. This work provides novel insights into the evolutionary plasticity and functional adaptation of viral genomes.

## Methods

### Retrieval of viral HSP70 sequences

A total of 74 viral HSP70 amino acid sequences were retrieved from the NCBI protein database using the search term ‘viruses’ and excluding partial sequences. To avoid redundancy, only one sequence per species was retained, except for *Cotonvirus japonicum*, Catovirus sp. ‘naegleriensis’ and Acanthamoeba castellanii mimivirus, which each encode two distinct HSP70 proteins of different lengths. Sequences were clustered using CD-HIT with a 95% identity threshold [16], resulting in 63 representative sequences for downstream analyses (Table S1). Host information was obtained from the Virus-Host DB (https://www.genome.jp/virushostdb/view/) or the literature. Additionally, 39 to 50 HSP70 sequences from plants, animals, protists, fungi, archaea and bacteria were randomly selected from the NCBI for phylogenetic comparison.

### Identification of HSP70 homologues in giant viruses

To identify HSP70 homologues in giant viruses, we created a custom HSP70 database from the retrieved sequences and formatted it using the makeblastdb tool. All protein-coding sequences from each giant virus genome were downloaded and queried against the HSP70 database using blastp [17]. Hits with *E*-values ≤10⁻³ were retained and manually verified. When full or near-complete genomes were available, gene positions and orientations were annotated. Pairwise amino acid identities were calculated using the Species Demarcation Tool [18] and visualized in RStudio 2024.09.1 (R version 4.3.3) using the libraries ‘ggplot2’ and ‘ggrepel’.

### Structural prediction and motif analysis

Protein structures were predicted using AlphaFold3 [19] via the online server (https://alphafoldserver.com/). Predicted structures were converted from CIF to PDB format using the mCIF-to-PDB converter (https://project-gemmi.github.io/wasm/convert/cif2pdb.html). Structural similarity matrices were generated using the DALI server [20]. Pairwise structural alignments were performed using the ‘align’ function in PyMOL (https://pymol.org) [21]. Structural similarity was assessed using DALI *z*-scores, with values >2 considered significant [22].

Motif discovery was conducted using the MEME suite [23] with the following parameters: any number of motif repetitions, a maximum of 20 motifs and motif widths between 10 and 100 aa. Motif positions were mapped onto 3D structures and visualized in PyMOL.

### Sequence alignment, phylogenetic and recombination analysis

Multiple sequence alignments were performed using muscle v5 with the -super5 option [24], followed by manual curation. Phylogenetic trees were constructed using the maximum-likelihood method implemented in IQ-TREE v2.2.0 [25]. The best-fit substitution model (LG+F+R5) was selected using ModelFinder [26]. For broader phylogenetic comparisons with cellular organisms, alignments were trimmed to remove poorly aligned regions, and trees were reconstructed using RAxML with the PROTGAMMALG model [27]. All trees were visualized using iTOL v5.0 (https//itol.embl.de). Recombination analyses were run using the genetic algorithm for recombination detection in the datamonkey server (https://www.datamonkey.org/) with the default parameters.

### Protein–protein interaction and selective pressure analyses

To investigate the interaction between the proteins, we used AlphaFold3 with the target proteins. The interaction significance was measured based on the pTM and ipTM scores, with a pTM >0.5 indicating that the overall predicted fold for the complex might be similar to the true structure. ipTM >0.8 represents confident high-quality predictions, whilst ipTM <0.6 suggests likely a failed prediction. ipTM values between 0.6 and 0.8 are a grey zone where predictions could be correct or incorrect.

To determine the selective pressures acting on the HSP70 and the p61 genes from *Closterovirus tristezae*, we used a branch-site unrestricted statistical test for episodic diversification, run in the datamonkey server. Multiple sequence alignments of the HSP70 and p61 genes were used as an input.

### Similarity network analysis

To investigate the functional relationships among viral HSP70 proteins and their similarity to cellular homologues, we constructed sequence similarity networks using the Enzyme Function Initiative-Enzyme Similarity Tool (EFI-EST) web server (https://efi.igb.illinois.edu) [28]. Viral HSP70 amino acid sequences were submitted to EFI-EST to generate pairwise sequence similarity networks based on blast alignment scores. Two alignment score thresholds were applied: a strict threshold of 35 to identify highly similar sequences and a relaxed threshold of 10 to capture more distant relationships.

To assess relationships with cellular HSP70s, we included representative sequences from plants, animals, fungi, protists, bacteria and archaea (ten species per group) and generated a network using both relaxed and strict thresholds.

The resulting networks were visualized using Cytoscape v3.9.1 [29].

## Results

### Distribution and abundance of viral HSP70s

**Fig. 1.**
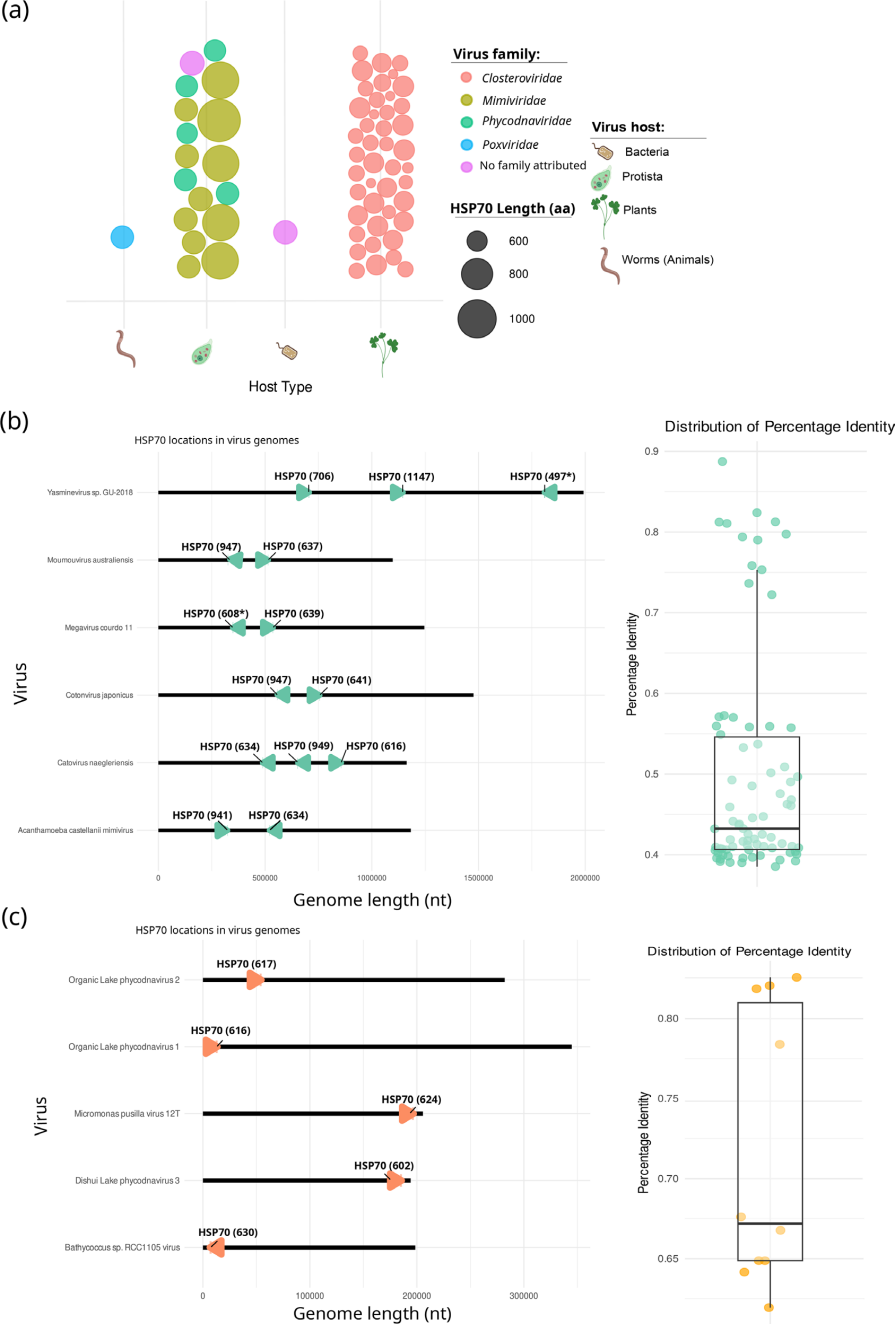
Abundance and genomic organization of HSP70 in viruses. (**a**) Abundance of HSP70 homologues in viruses categorized by protein size, host organism and viral family. (**b**) Genomic organization of HSP70 in *Imitervirales* members: gene location, length, strand orientation and pairwise identity. (**c**) Genomic organization in *Algavirales* members. Fragmented HSP70s are marked with an asterisk (*).

We retrieved 63 viral HSP70 sequences from the NCBI protein database, with lengths ranging from 533 to 1,147 aa (Fig. 1a, Table S1, available in the online Supplementary Material). These sequences were primarily from two viral classes: *Alsuviricetes* (ssRNA viruses infecting plants) and *Megaviricetes* (giant dsDNA viruses infecting protists). *Alsuviricetes*, specifically members of the *Closteroviridae* family, accounted for 68% of the sequences, with HSP70 lengths between 533 and 606 aa. *Megaviricetes* included viruses from the orders *Algavirales* and *Imitervirales* and families *Phycodnaviridae* and *Mimiviridae*, respectively. *Phycodnaviruses* (8%) infect algae, whilst mimiviruses (20%) infect amoebae.

Two additional sequences were identified from less-represented classes: an *Acinetobacter* phage MD2-2021a (CAH1093665; *Caudoviricetes*) and *Poxvirus euperipatoides* 37252 (DBA47115, unclassified *Entomopoxvirinae*), infecting bacteria and velvet worms, respectively. blastp analysis for the *Acinetobacter* phage HSP70 yielded homologues to the DnaK chaperone from 25 related bacteria (>96% identity), whilst no additional homologues were found for the poxvirus sequence, suggesting a possible misannotation.

### Copy number variation in *Megaviricetes*

Among members of the *Imitervirales* order, several viruses encoded multiple HSP70 copies. Acanthamoeba castellanii mimivirus, Catovirus sp. ‘naegleriensis’ and *C. japonicum* each encoded two HSP70s (~600 and ~900 aa, respectively). Other imiterviruses, such as Bandra megavirus, Hyperionvirus sp. and Yasminevirus sp., encoded single large HSP70s (>900 aa) (Table S1).

To investigate this further, we analysed complete genomes of viruses in *Imitervirales*. Most encoded two HSP70s, except Catovirus sp. ‘naegleriensis’ and Yasminevirus sp., which had three (Fig. 1b, Table S2). These genes were randomly distributed across both DNA strands and showed no correlation with protein length. Pairwise amino acid identity ranged from 39 to 88%, with small HSP70s (<655 aa) showing higher similarity (>80%) among *Moumouvirus australiense* and Megavirus courdo 11 and Acanthamoeba castellanii mimivirus (Fig. S1).

In contrast, members of the *Algavirales* order encoded only one HSP70 per genome, all small (602–624 aa), located primarily on the sense strand (Fig. 1c, Table S3). Pairwise identity among these proteins ranged from 61 to 81% (Fig. 1c). Members of the *Pimascovirales* order did not encode for any HSP70.

### Gene structure and expression patterns

Most viral HSP70s were encoded by single-exon genes. However, exceptions were observed in Megavirus courdo 11 and Yasminevirus sp. In megavirus courdo 11, one HSP70 gene (639 aa) contained an intron, whilst another (609 aa) appeared split across two intergenic regions, producing two immature proteins that aligned structurally to form a complete HSP70 (Fig. S2A, B). Similarly, Yasminevirus sp. encoded two gene fragments separated by an unrelated ORF, which together formed a full-length HSP70 (Fig. S2C). These findings suggest alternative splicing or gene fragmentation mechanisms in some *Imitervirales*.

### Structural and motif diversity

**Fig. 2.**
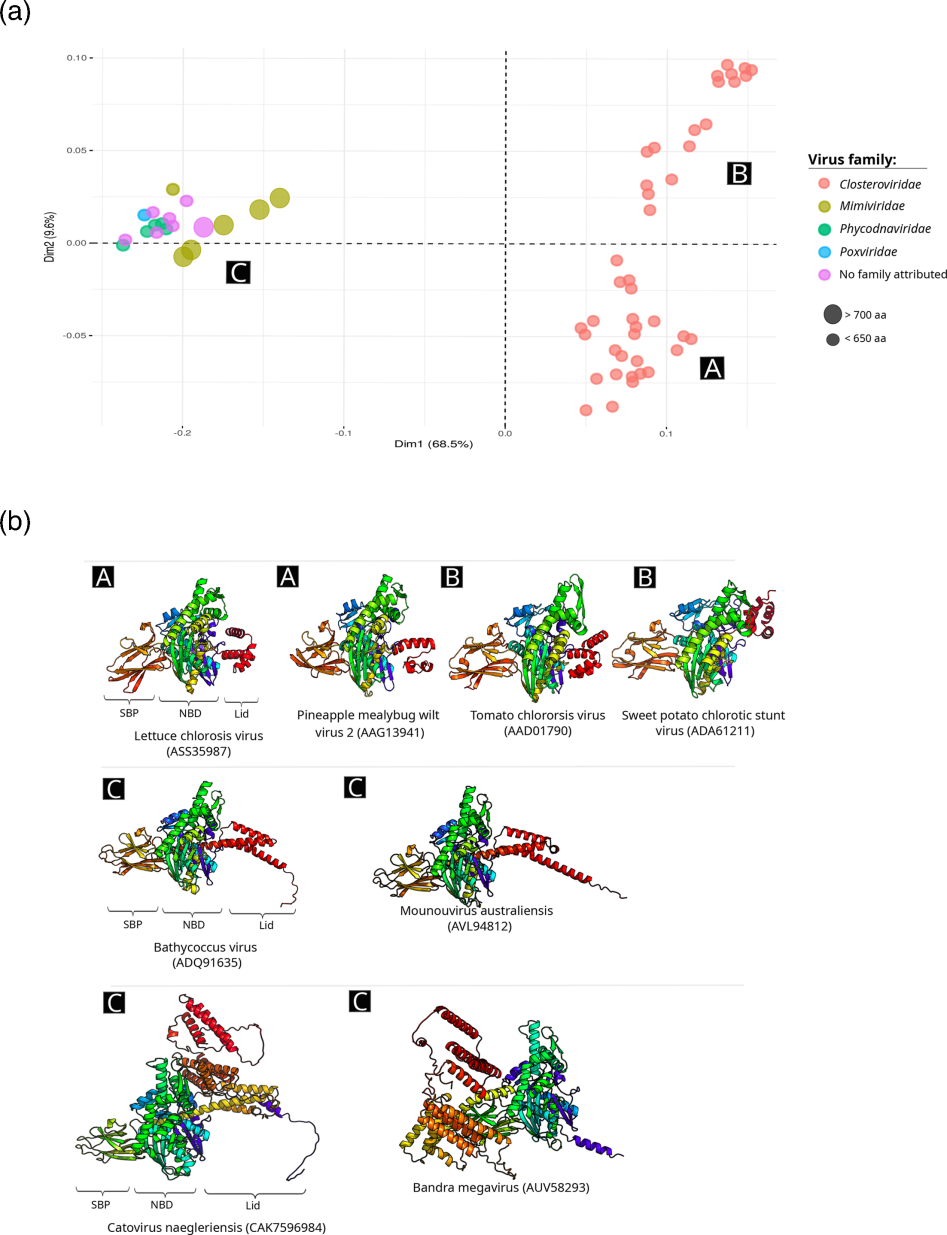
Structural diversity of viral HSP70 proteins. (**a**) CA based on structural similarity, with proteins labelled by virus family and size. (**b**) Representative 3D structures from each CA cluster, coloured from N-terminus (blue) to C-terminus (red).

To assess structural diversity, we predicted 3D models of all viral HSP70s and performed correspondence analysis (CA) based on structural similarity. The CA revealed two major clusters (Fig. 2a): one comprising viruses with ssRNA genomes from the *Closteroviridae* with high structural dispersion and another comprising viruses with dsDNA genomes with more compact structures. The main structural differences were observed in the SBD, which was shorter and more variable in viruses with ssRNA genomes (Fig. 2b). Phylogenetic analysis of closteroviruses showed a distribution according to the virus genera, with some exceptions: HSP70 from plant-associated crinivirus 1 which clustered with HSP70 from closteroviruses, blackberry dwarf-associated virus HSP70s clustered with the ones from ampeloviruses, croton golden spot-associated virus and plant-associated closterovirus 2 which clustered with criniviruses (Fig. S3A). Recombination analyses inferred at least three breakpoints (Fig. S3B).

**Fig. 3.**
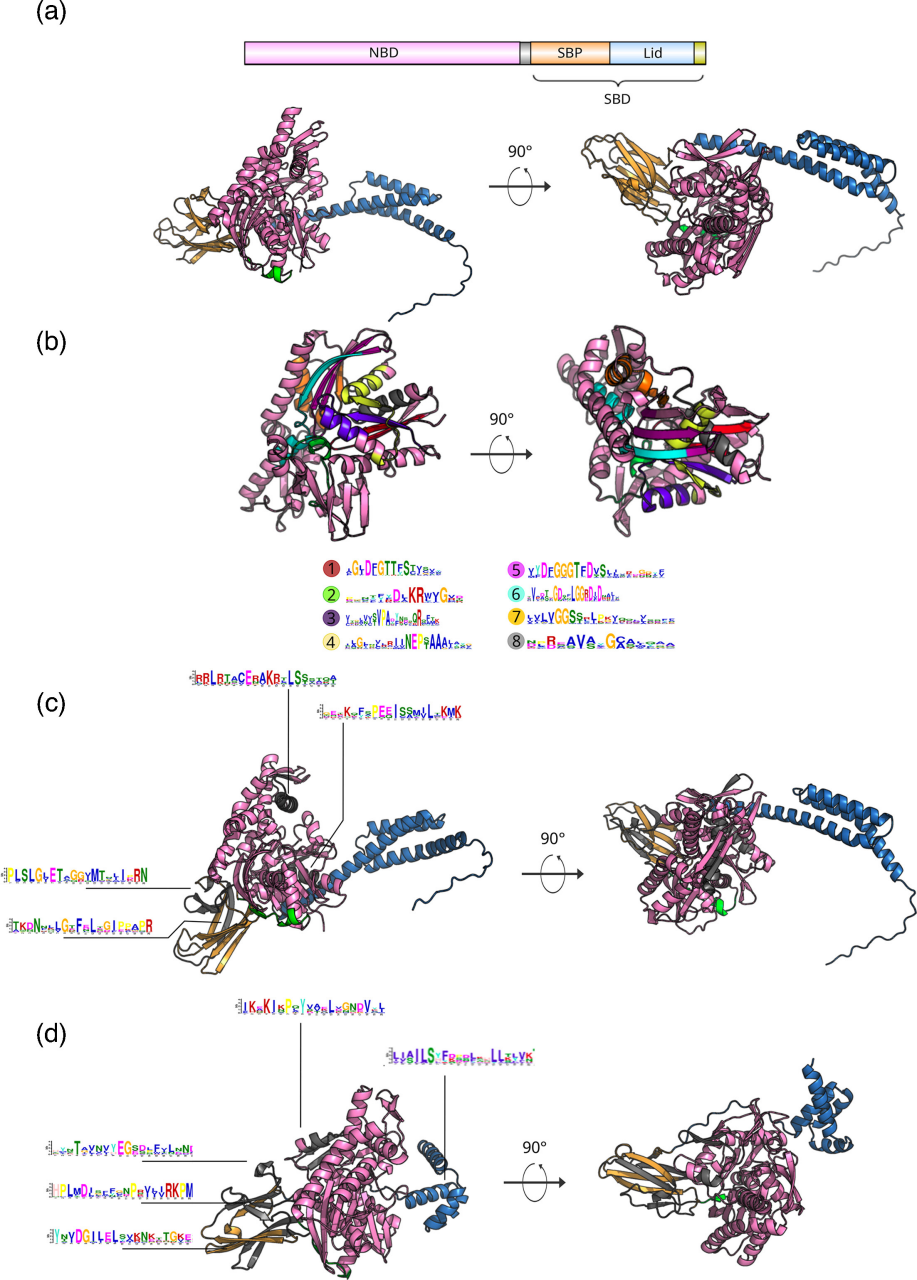
Domain architecture and motifs in viral HSP70s. (**a**) Schematic of HSP70 domains from Bathycoccus virus (GenBank: BADQ91635), showing the NBD (in pink) and SBD, including the lid (in blue) and substrate-binding pocket (SBP, in orange) and the EEVD motif (in chartreuse). (**b**) Positions of conserved motifs (1–8) within the NBD across all viral HSP70s. Full motif sequences are listed in Table S4. (**c**) Motifs unique to dsDNA viruses (giant viruses), mapped onto the HSP70 from Bathycoccus virus (BADQ91635). (**d**) Motifs specific to ssRNA closteroviruses, illustrated using the HSP70 from Areca palm velarivirus 1 (*Velarivirus arecae*; YP_009140434).

Motif analysis identified eight conserved motifs (1–8) present in all viral HSP70s, primarily located in the NBD (Fig. 3a, b, Table S4). Four additional motifs (9, 10, 13 and 17) were specific to dsDNA viruses, located in both the SBD and NBD (Fig. 3c). Conversely, four motifs unique to members of *Closteroviridae* were longer (26–50 aa) and variably distributed (Fig. 3d). Notably, all *Closteroviridae* HSP70s lacked the canonical EEVD motif at the C-terminus.

### HSP70 from closteroviruses reveals a specific association with the p61 protein

**Fig. 4.**
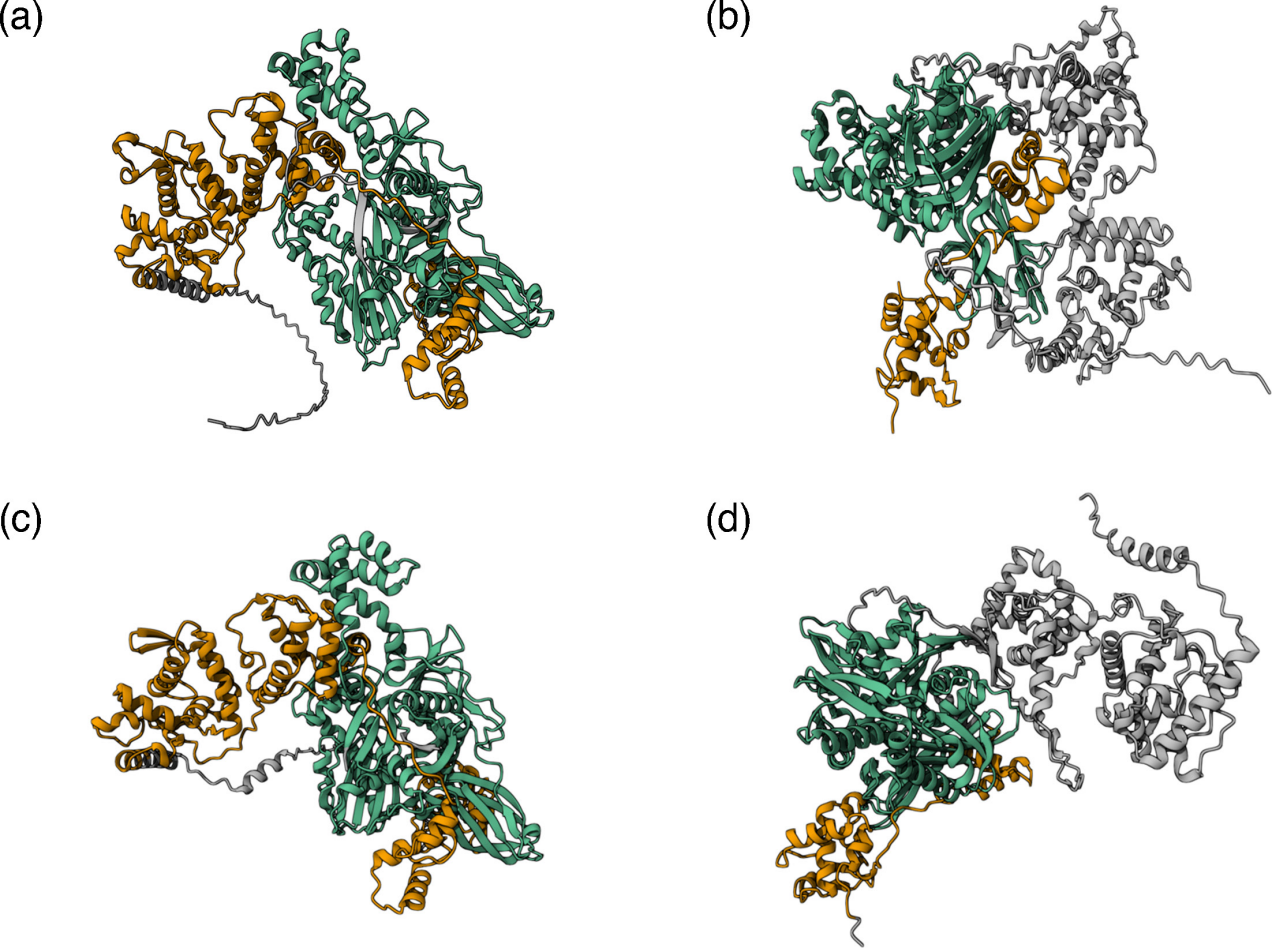
Protein–protein interaction of the p61 with the different HSP70. (**a**) The interaction of the p61 and the HSP70 from *C. tristezae* (NC_001661) and (**b**) the interaction of the p61 from *C. tristezae* with the HSP70 from olive virus P (*Olivavirus*; WLJ59777). (**c**) The interaction of the p61 (YP_009058933) and HSP70 (A0A088MGX5) from *Closterovirus rosafolium*. (**d**) The interaction of the p61 of *C. rosafolium* with the HSP70 from olive virus P. The coloured chains indicate the interacting domains from the p61 (orange) and the HSP70 (green), whilst the grey chains mark non-interacted domains.

We further explored the impact of these observed modifications at the protein interaction level. HSP70 from closteroviruses interacts with other virus proteins like the minor coat protein (CPm), p61 and p6 to form the tail complex that is indispensable for the cell-to-cell movement [30]. Our *in silico* analyses revealed a significant interaction between p61 and HSP70 of members of the species *C. tristezae* (ipTM=0.84; pTM=0.79) (Fig. 4a), whilst no interaction was observed between HSP70 and other proteins (data not shown).

This interaction was completely hampered when substituting the HSP70 from *C. tristezae* by HSP70 from the host (*Citrus sinensis*; KAH9715890) (ipTM=0.17; pTM=0.4) or from dsDNA viruses (Catovirus sp. ‘naegleriensis’; CAK7596788) (ipTM=0.17; pTM=0.39) and partially hampered when using a divergent HSP70 from olive virus P (genus *Olivavirus*; WLJ59777) but with low confidence (ipTM=0.48; pTM=0.52) (Fig. 4b). Similar results were obtained from the interaction of the p61 and HSP70 from *Closterovirus rosafolium* (YP_009058933) (ipTM=0.82; pTM=0.8) (Fig. 4c), with HSP70 from plant (*Ziziphus jujuba*; XP_048331791) (ipTM=0.31; pTM=0.41), with the HSP70 from dsDNA viruses (Catovirus sp. ‘naegleriensis’) (ipTM=0.17; pTM=0.39) and with the HSP70 from olive virus P but with low confidence (ipTM=0.45; pTM=0.53) (Fig. 4d).

We next investigated the selective pressures acting on the HSP70 and the p61 genes. We used 65 sequences from complete * C. tristezae*, and we found evidence of episodic diversifying selection in eight sites in the HSP70 (*P*<0.001; Fig. S4) and eight sites in the p61 (*P*<0.001; Fig. S5).

### Phylogenetic relationships and host associations

**Fig. 5.**
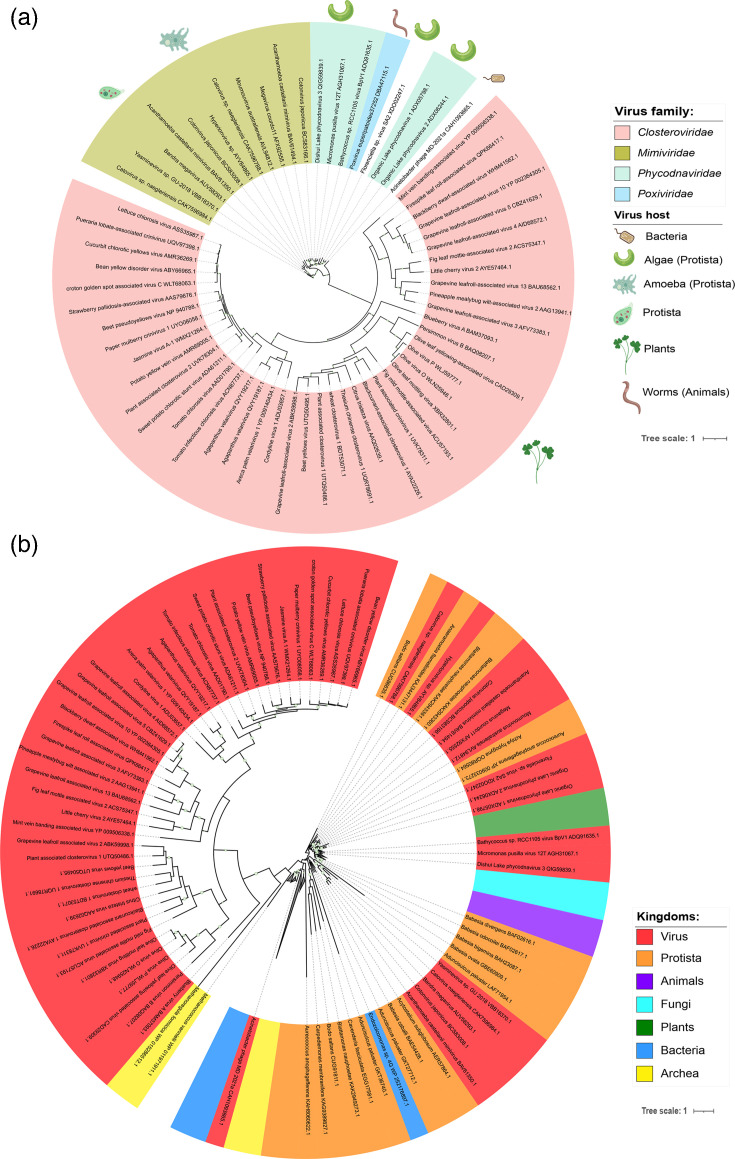
Phylogenetic tree of HSP70 from viruses and other organisms. (**a**) Phylogenetic tree of viral HSP70 constructed using IQ-TREE v2 with the LG+F+R5 substitution model. (**b**) Phylogenetic relationships between viral and cellular HSP70s (plants, fungi, protists, bacteria and archaea), constructed using RaxML with the PROTGAMMALG model. Bootstrap values >70% (1,000 replicates) are shown as green dots. Leaves of HSP70 from animals, plants, archaea, bacteria and fungi were collapsed. The full tree is shown in Fig. S6 in the supplementary material.

Phylogenetic analysis of viral HSP70s revealed distinct clades (Fig. 5a). Firstly, *Closteroviridae* HSP70 formed a well-supported, diverse clade with long branch lengths, indicating fast evolutionary diversification. Interestingly, this clade does not relate to plant HSP70s, suggesting an ancestral and independent origin. Secondly, *Megaviricetes* sequences formed three groups: one of large mimiviral HSP70s (900–1,100 aa), a second of smaller mimiviral HSP70s (~600 aa) and a third combining phycodnaviruses and poxvirus sequence.

To explore evolutionary relationships between host and viral HSP70s, we constructed a broader phylogeny including HSP70s from bacteria, archaea, fungi, protists, plants and animals (Fis 5b and S6). Viral HSP70s did not form a monophyletic group. Instead, *Closteroviridae* HSP70 formed a monophyletic cluster clearly distinct from all cellular taxa, whilst *Megaviricetes* HSP70s formed different clusters always related to different protist sequences, suggesting multiple events of HGT from host to ancestral viruses.

### Functional network analysis reveals divergent evolutionary paths of viral HSP70s

**Fig. 6.**
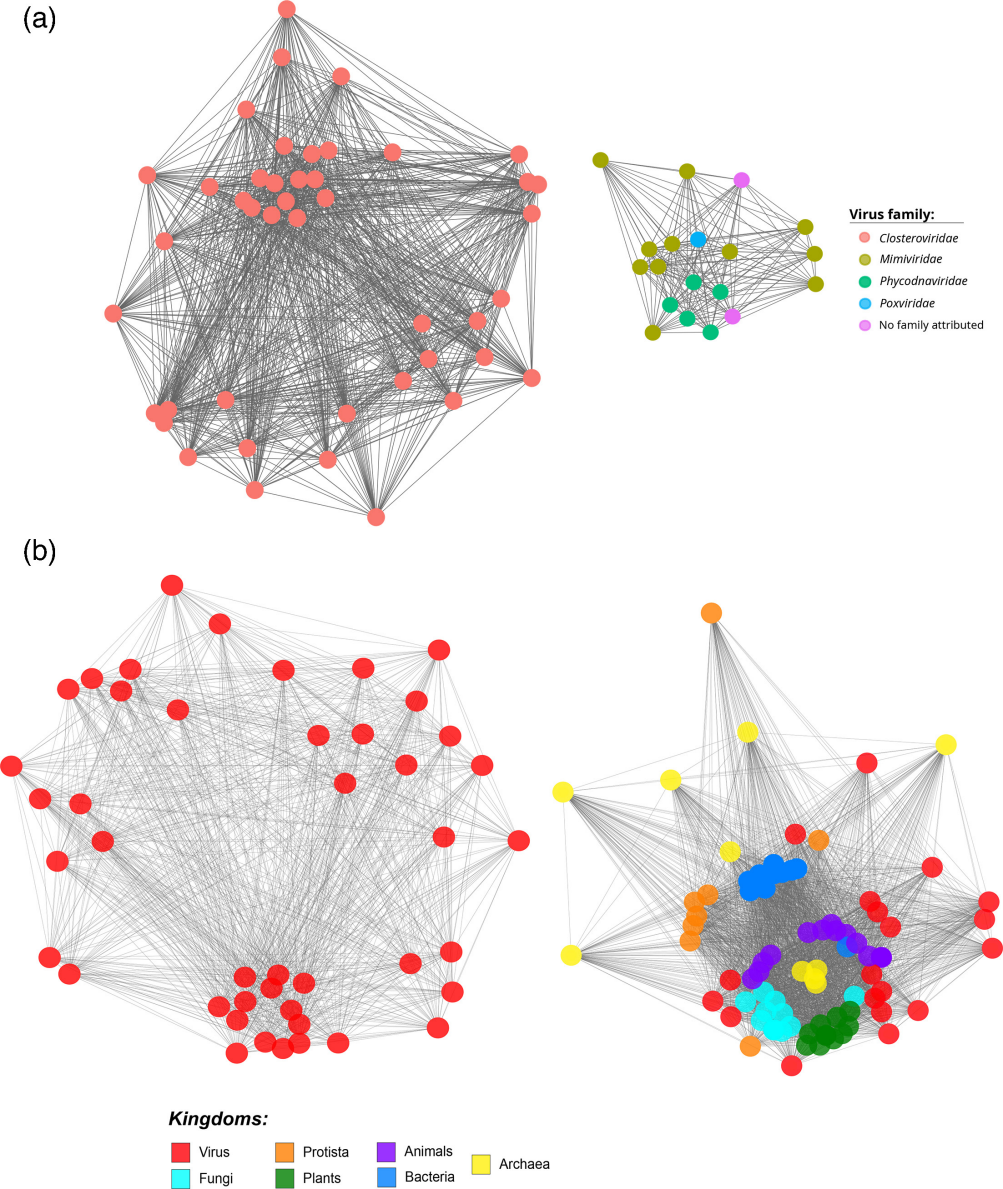
Sequence similarity network of viral and cellular HSP70 proteins generated using the EFI-EST tool. (**a**) Network of viral HSP70s constructed using a strict alignment score threshold of 35. Two completely disconnected clusters are observed: one composed of HSP70s from ssRNA viruses (*Closteroviridae*) and the other from dsRNA viruses, indicating strong sequence divergence. (**b**) Network including HSP70s from viruses and representative cellular organisms (ten species each from plants, animals, fungi, protists, bacteria and archaea), constructed using the strict threshold (35). HSP70s from ssRNA viruses form a distinct, isolated cluster, whilst those from dsDNA viruses integrate with cellular HSP70s, mainly from protists.

To further explore the functional relationships among viral HSP70 proteins, we constructed sequence similarity networks using the EFI-Enzyme Similarity Tool (EFI-EST) [28]. At a strict alignment score threshold of 35, the network of viral HSP70s (Fig. 6a) resolved into two completely disconnected clusters: one composed exclusively of HSP70s from closteroviruses (ssRNA genomes) and the other comprising HSP70s from dsDNA viruses. This separation further supports the hypothesis of distinct evolutionary origins for these groups. When the threshold was relaxed to 10 (Fig. S7A), all viral HSP70s formed a single connected network; however, the spatial separation of nodes still indicated two major subgroups, consistent with functional divergence.

To assess the relationship between viral and cellular HSP70s, we generated a network including representative sequences from major taxonomic groups (plants, animals, fungi, protists, bacteria and archaea) along with all viral HSP70s, using the strict threshold (Fig. 6b). In this network, HSP70s from ssRNA viruses again formed a separate, isolated cluster, whilst those from dsDNA viruses integrated with cellular sequences, particularly those from protists, further supporting HGT from host to virus. When the threshold was relaxed to 10 (Fig. S7B), again all viral HSP70s formed a single connected network; however, the spatial separation of nodes still indicated two major subgroups, consistent with functional divergence. These network-based findings reinforce our structural and phylogenetic analyses, highlighting both the evolutionary independence of ssRNA viral HSP70s and the host-derived origins of their dsDNA counterparts.

## Discussion

This study provides the first comprehensive analysis of HSP70 homologues encoded by viruses, revealing their limited but diverse presence across viral taxa and offering new insights into their structural features, gene organization and evolutionary origins. Our results confirm that viral HSP70s are not widespread but are instead restricted to specific lineages, primarily ssRNA viruses from the *Closteroviridae* family and dsDNA viruses from *Mimiviridae* and *Phycodnaviridae*. This restricted distribution suggests that HSP70 acquisition is not a universal viral trait but rather a lineage-specific adaptation, likely driven by ecological or functional pressures. The presence of HSP70s in *Closteroviridae* has been previously reported [13,14], but our study expands this to include dsDNA viruses, particularly those infecting protists.

We observed that whilst most viruses encode a single HSP70 gene, members of *Imitervirales* often harbour two or three copies. These genes are randomly distributed across the genome and located on both strands, suggesting independent insertion events. In some cases, such as Megavirus courdo 11 and Yasminevirus sp., we identified atypical gene architectures, including intron-containing genes and fragmented ORFs that still produce structurally coherent proteins. However, it is not clear whether these proteins retain their function, or if the loss of function has driven the virus to hijack other functional HSP70 from their hosts to fulfil that role. These findings suggest the presence of virus-specific mechanisms such as alternative splicing or post-translational assembly [31,34], highlighting the remarkable genome plasticity in large dsDNA viruses.

Structural modelling and CA revealed two major HSP70 clusters: one comprising ssRNA viruses and another comprising dsDNA viruses. The ssRNA virus HSP70s exhibited greater structural variability, particularly in the SBD, consistent with their higher evolutionary rates [35]. Recombination analyses revealed frequent reassortment events, shedding light on its implication on the observed diversity. In contrast, dsDNA virus HSP70s were more structurally conserved. Motif analysis identified both universally conserved motifs and lineage-specific ones. Notably, all *Closteroviridae* HSP70s lacked the canonical EEVD motif, which is typically involved in co-chaperone interactions [2,4], suggesting functional divergence from their cellular counterparts.

Closteroviruses require tail assembly for the cell-to-cell movement [36]. The CPm attaches to specific loops in the 5′ UTR and covers the 5′ region of the virus. This process requires the attachment of the HSP70 and the p61. The movement process also requires the p6 protein, which acts as a single-span transmembrane protein that resides in endoplasmic reticulum [37]. We were not able to determine these interactions *in silico* except for the HSP70 and p61. This would probably indicate the involvement of additional physical or chemical cell components. However, the HSP70 and the p61 interaction shed light on the importance of the HSP70 specificity to comply with its role, and how its substitution with other HSP70 either from the host, dsDNA viruses or other related ssRNA viruses may interrupt this interaction. Despite this interaction specificity, both genes encoding these two proteins are under diversifying selection.

Phylogenetic analyses revealed that viral HSP70s do not form a monophyletic group. Instead, they are scattered across the tree of life, with dsDNA virus HSP70s clustering near their protist hosts and ssRNA virus HSP70s forming a distinct, highly divergent clade, unrelated to their plant hosts. These patterns strongly suggest multiple independent HGT events from cellular organisms to viruses. The close relationship between *Megaviricetes* HSP70s and protist sequences supports the hypothesis of host-to-virus HGT [38], whilst the long branch lengths of *Closteroviridae* HSP70s may reflect an ancient acquisition followed by rapid evolution [14].

To further explore functional relationships among viral HSP70s, we constructed sequence similarity networks using the EFI-EST tool. At a strict alignment score threshold, viral HSP70s formed two completely disconnected clusters: one composed of plant ssRNA viruses and the other of dsDNA viruses. This separation reinforces the deep sequence divergence between these groups. When the threshold was relaxed, all viral HSP70s formed a single connected network, but the spatial separation of nodes still reflected two major subgroups, consistent with functional divergence. When cellular HSP70s were included in the network (using the strict threshold), ssRNA viral HSP70s again formed an isolated cluster, whilst dsDNA viral HSP70s integrated with cellular sequences, particularly from protists, further supporting the hypothesis of HGT from host to virus. These network-based findings align with our structural and phylogenetic results, providing additional evidence for the distinct evolutionary trajectories of viral HSP70s.

**Fig. 7.**
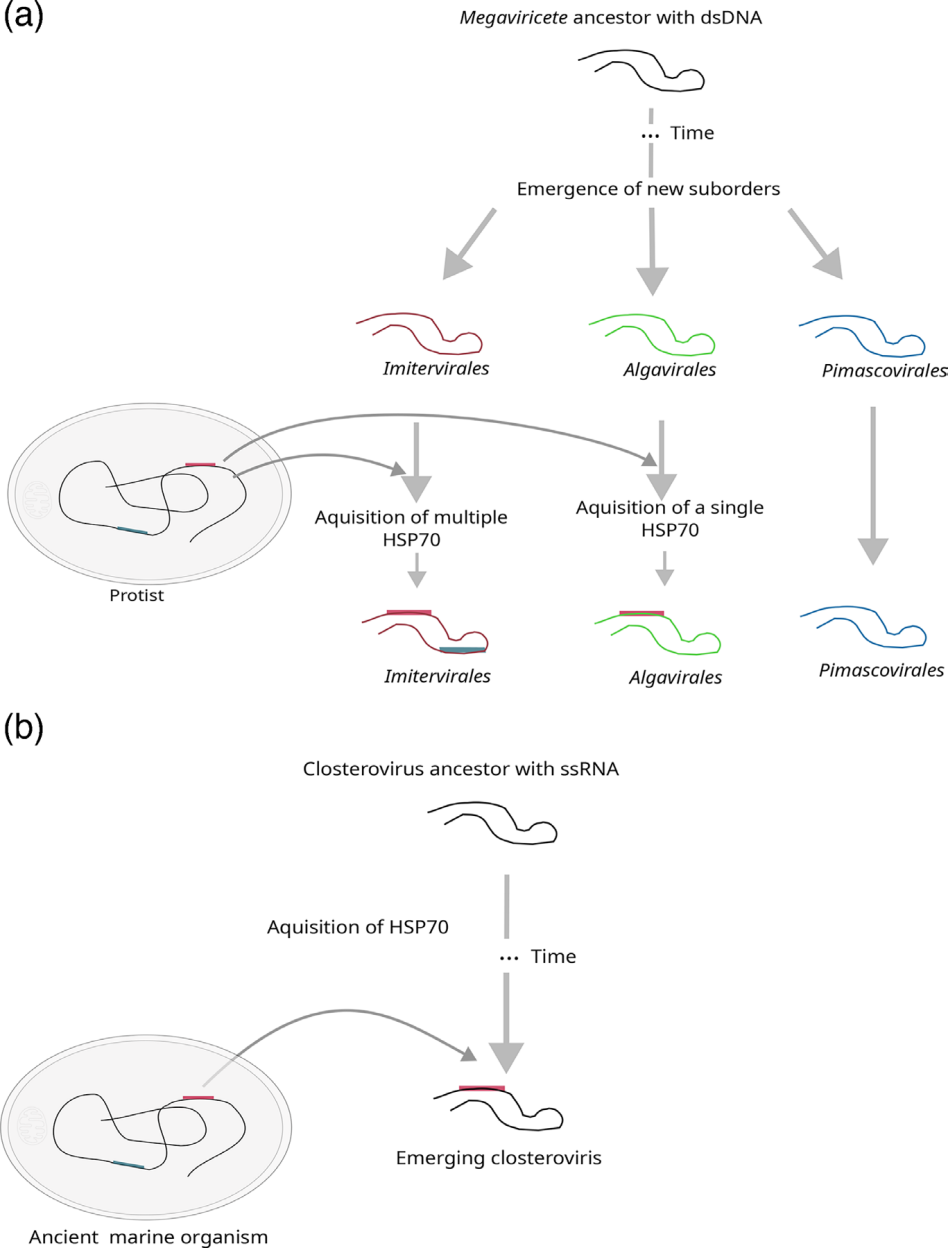
Proposed evolutionary scenarios for the acquisition of the HSP70 genes in viruses. (**a**) The divergence of the *Megaviricetes* ancestor into three lineages *Algavirales*, *Imitervirales* and *Pimascovirales,* followed by independent and multiple acquisition of the HSP70 in *Imitervirales,* a single acquisition in *Algavirales* and no acquisition in *Pimascovirales*. (**c**) Separate acquisition in closteroviruses from an unknown source followed by extensive modifications through mutation, deletion and recombination events.

Based on our findings, we propose an evolutionary scenario for dsDNA viruses on which *Megaviricetes* ancestors have diverged into the lineages *Imitervirales*, *Algavirales* and *Pimascovirales*, followed by independent and multiple acquisition with or without loss of HSP70 in *Imitervirales*, a single acquisition in *Algavirales* and no acquisition in *Pimascovirales* (Fig. 7a). For ssRNA viruses, the data support a single, ancient acquisition from an ancestor marine organism, followed by extensive divergence (Fig. 7b), coupled with protein size reduction to play a crucial role in virus encapsidation and movement. Our results suggest that plant closteroviruses probably have a marine origin and were infecting marine organisms before specializing in land plants.

This study provides foundational insights into the evolution and diversity of viral HSP70s. Future experimental work is needed to determine the functional roles of these proteins in viral replication, host interaction and stress response, particularly in dsDNA viruses where their biological significance remains largely unexplored.

## Supplementary material

10.1099/jgv.0.002242 Uncited Supplementary Material 1.

10.1099/jgv.0.002242 Uncited Supplementary Material 2.

## Abbreviations

EFI-EST: Enzyme Function Initiative-Enzyme Similarity Tool
HGT: horizontal gene transfer
HSP: heat shock protein
HSP70: heat shock protein 70
NBD: nucleotide-binding domain
SBD: substrate-binding domain
