# Supplementary material for: Multiple origins and functions: evolutionary pathways of HSP70 proteins in viruses
Source: J Gen Virol. 2026 Mar 10;107(3):002242. doi: 10.1099/jgv.0.002242 (PMC12978162; doi:10.1099/jgv.0.002242)
Supplement: Uncited Supplementary Material 1. [file jgv-107-02242-s001.pdf]

Figure S1

A

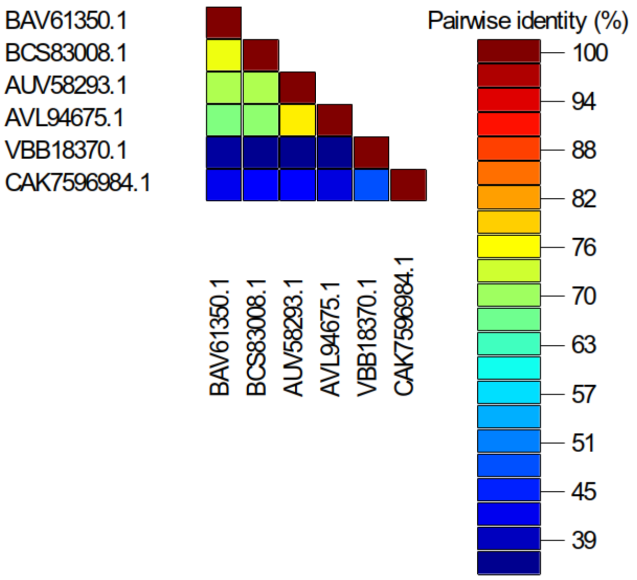

B

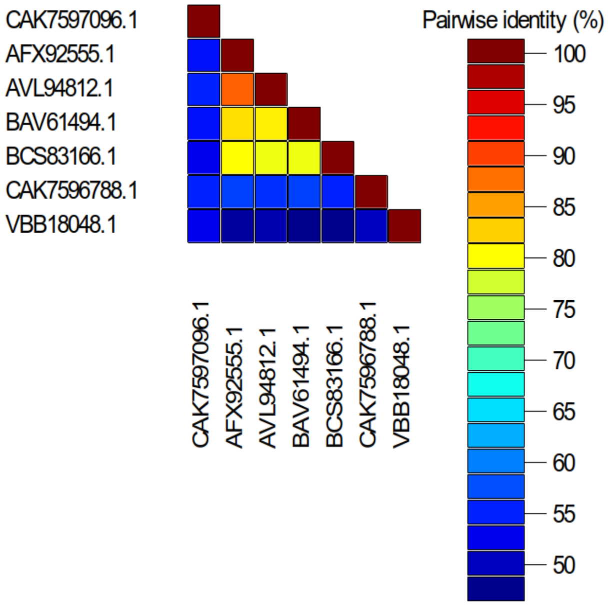

Figure S1. (A) Pairwise amino acid identity among large HSP70 proteins (> 900 amino acids) from Imitervirales members: Acanthamoeba castellanii mimivirus (Genbank accession: BAV61350), Bandra megavirus (GenBank: AUV58293), Catovirus naegleriensis (GenBank: CAK7596984), Cotonvirus japonicus (GenBank: BCS83008), Moumouvirus australiensis (GenBank: AVL94675), and Yasminevirus (GenBank: VBB18370). (B) Pairwise identity among small HSP70 proteins (< 650 amino acids) from Imitervirales members: A. castellanii mimivirus (GenBank: BAV61494), C. naegleriensis (GenBank: CAK7597096, CAK7596788), C. japonicus (GenBank: BCS83166), Megavirus courdo 11 (GenBank: AFX92555), M. australiensis (GenBank: AVL94812), and Yasminevirus (GenBank: VBB18048).

Figure S2

A

Megavirus courdo 11

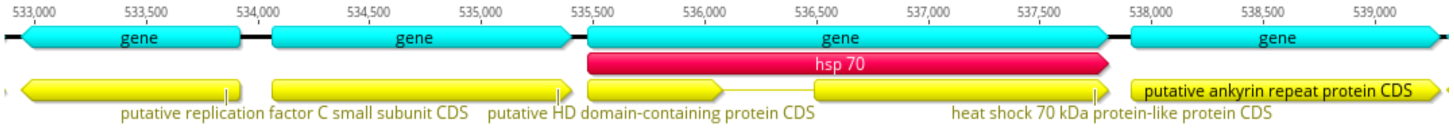

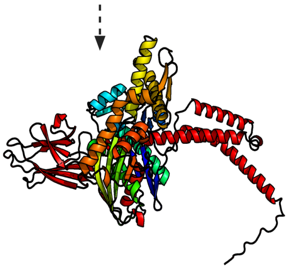

B

Megavirus courdo 11

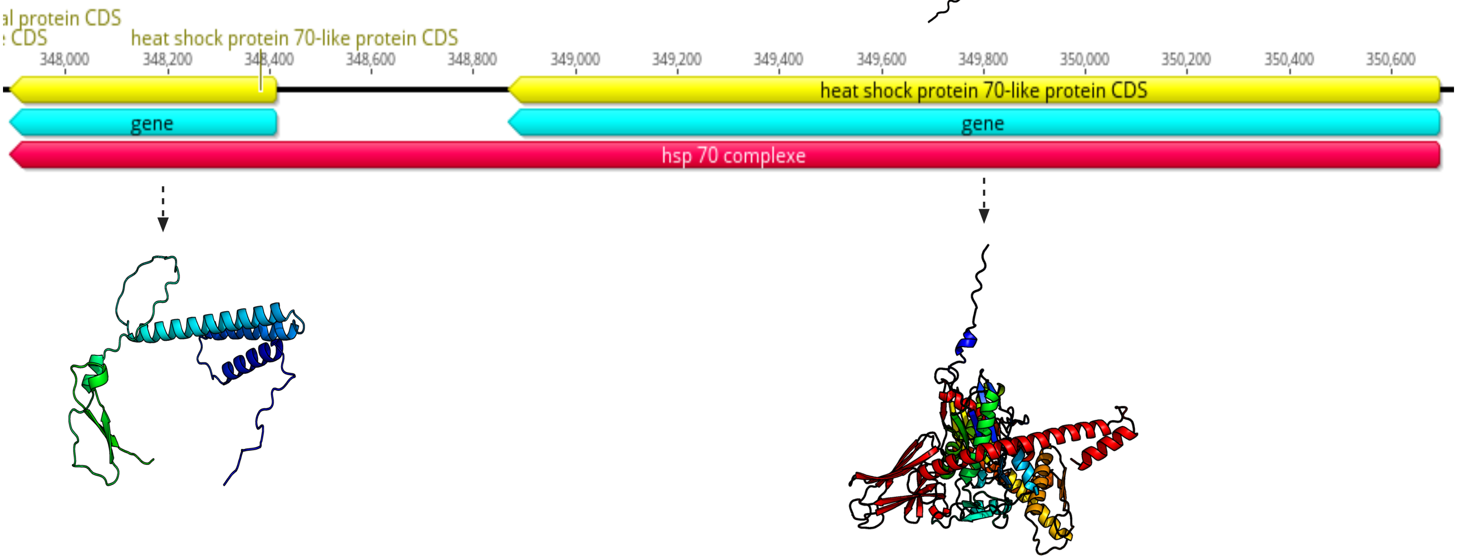

C

Yasmenivirus

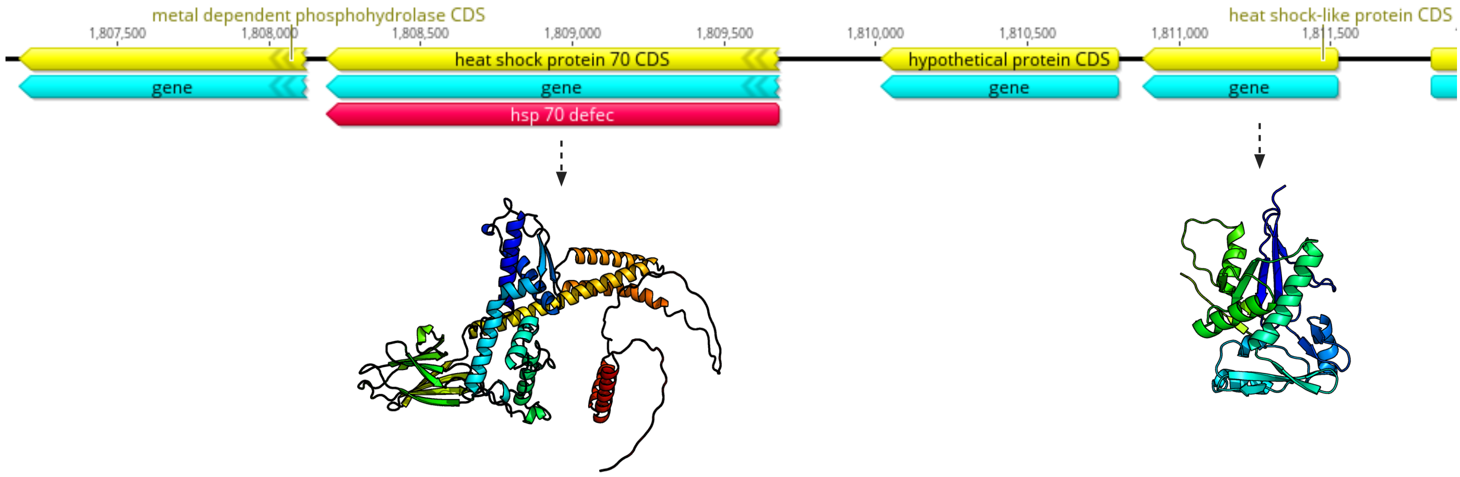

Figure S2. (A) HSP70 gene from Megavirus courdo 11 showing expression via alternative splicing. (B) Two adjacent HSP70 genes from M. courdo 11, separated by an intron-like region. The predicted 3D structures of their translation products align to form a complete HSP70 protein. (C) Fragmented HSP70 genes in Yasminevirus, separated by an unrelated intervening ORF. The combined translation products yield a full-length HSP70 protein.

Figure S3

A

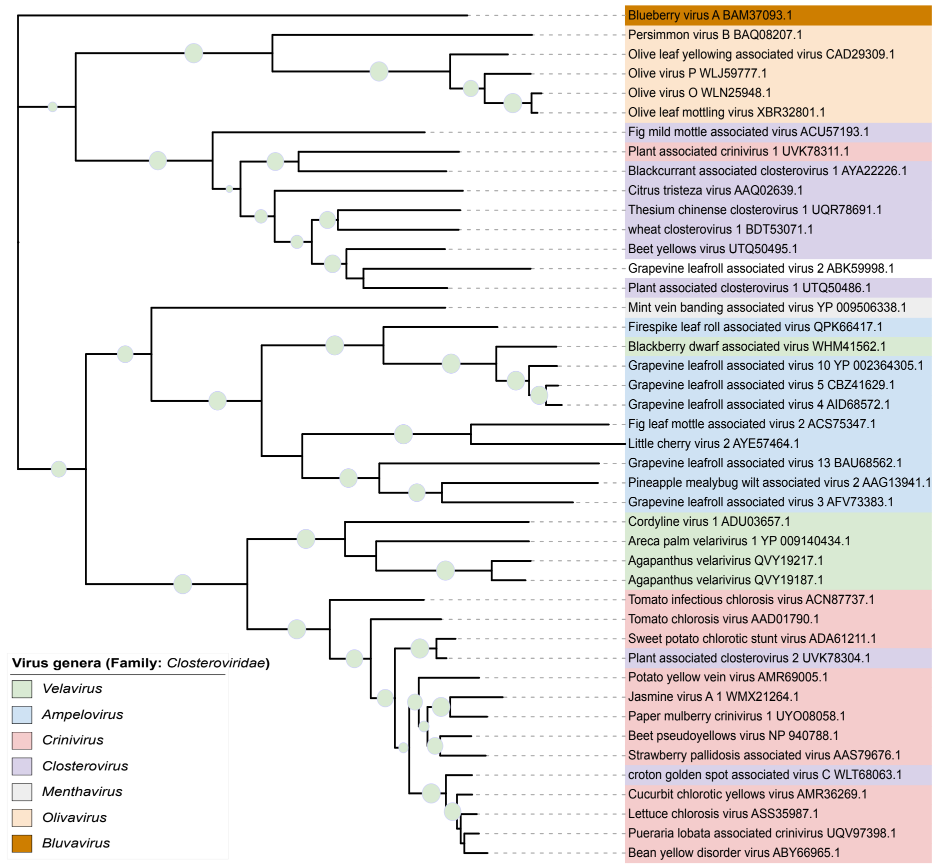

B

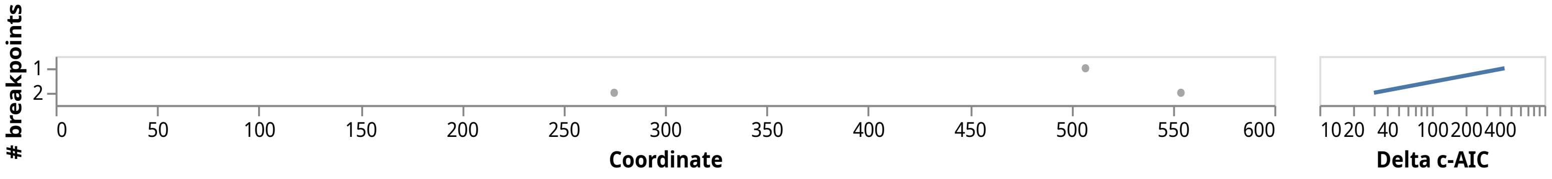

Figure S3. Phylogenetic relationships and recombination analyses of the HSP70 from ssRNA closteroviruses. (A) The phylogenetic tree includes sequences from the different genera from the Closteroviridae family, constructed using IQ-TREE v2 with the LG+F+R5 model. Bootstrap values > 70% (1000) replicates) are shown as green circles in the branches. (B) Recombination analyses output indicating the best placement of the breakpoints inferred by the GARD algorithm for each number of breakpoints considered (left panel), and the improvement in the cAIC score between successive breakpoint numbers (log scale).

Figure S4

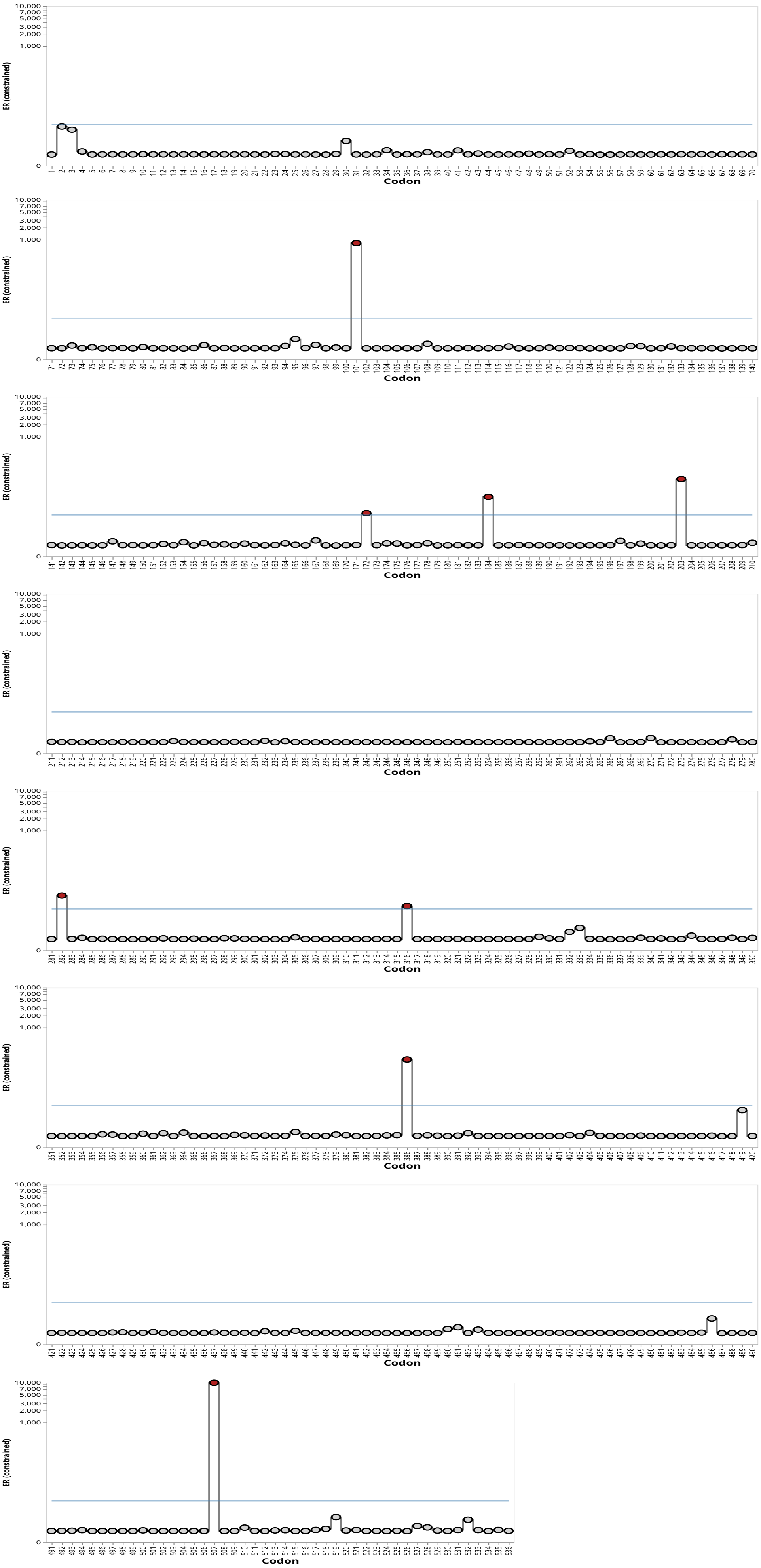

Figure S4. Evidence ratios (site level likelihood ratios) for ω>1 in the heat shock protein 70 gene from Closterovirus tristezae virus, comparing the unrestricted model with the model where max(ω) = 1, and all other parameters are kept at their maximum likelihood values. Solid line indicate the significance threshold.

Figure S5

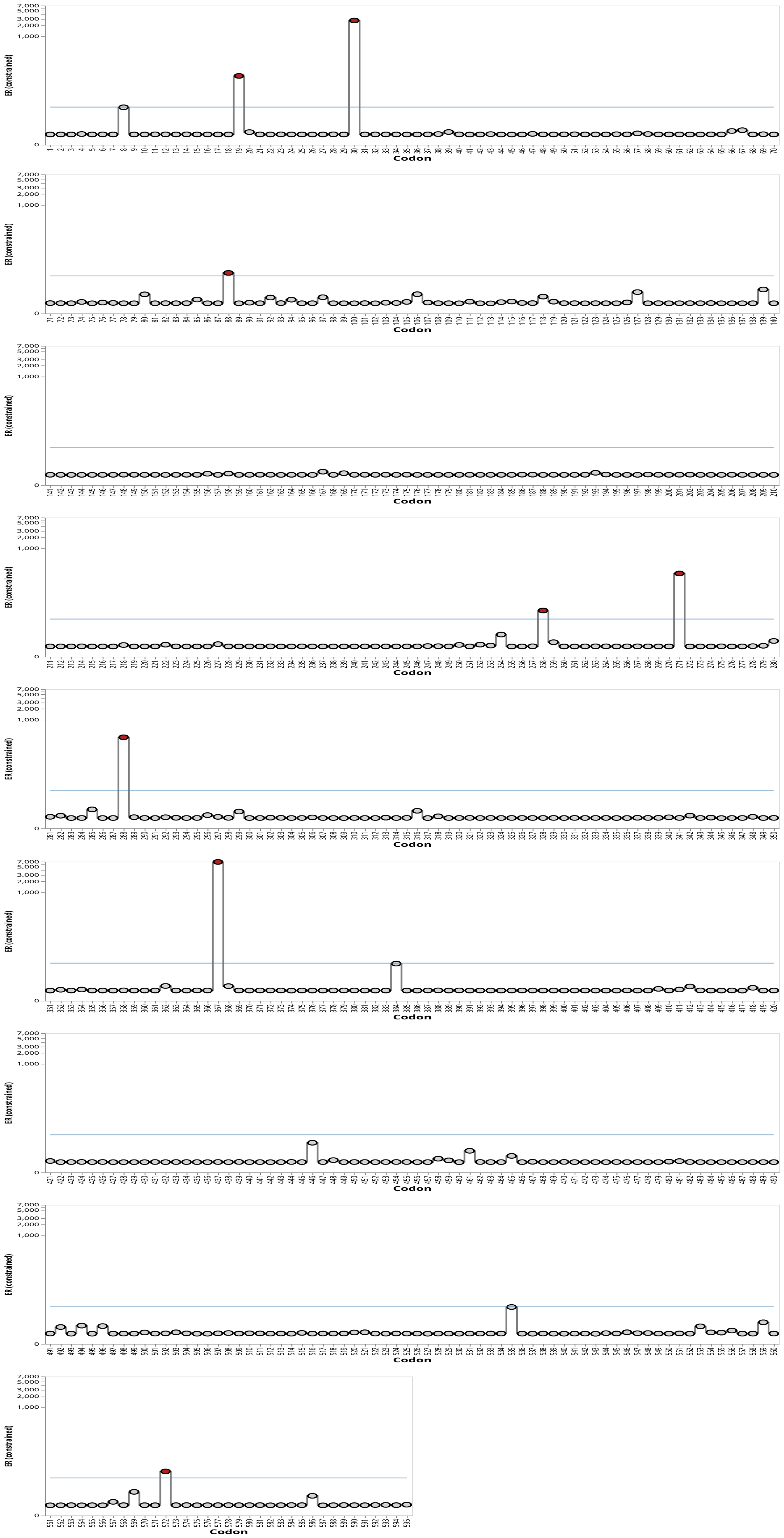

Figure S5. Evidence ratios (site level likelihood ratios) for ω>1 in the p61 gene from Closterovirus tristezae, comparing the unrestricted model with the model where max(ω) = 1, and all other parameters are kept at their maximum likelihood values. Solid line indicate the significance threshold.

Figure S6

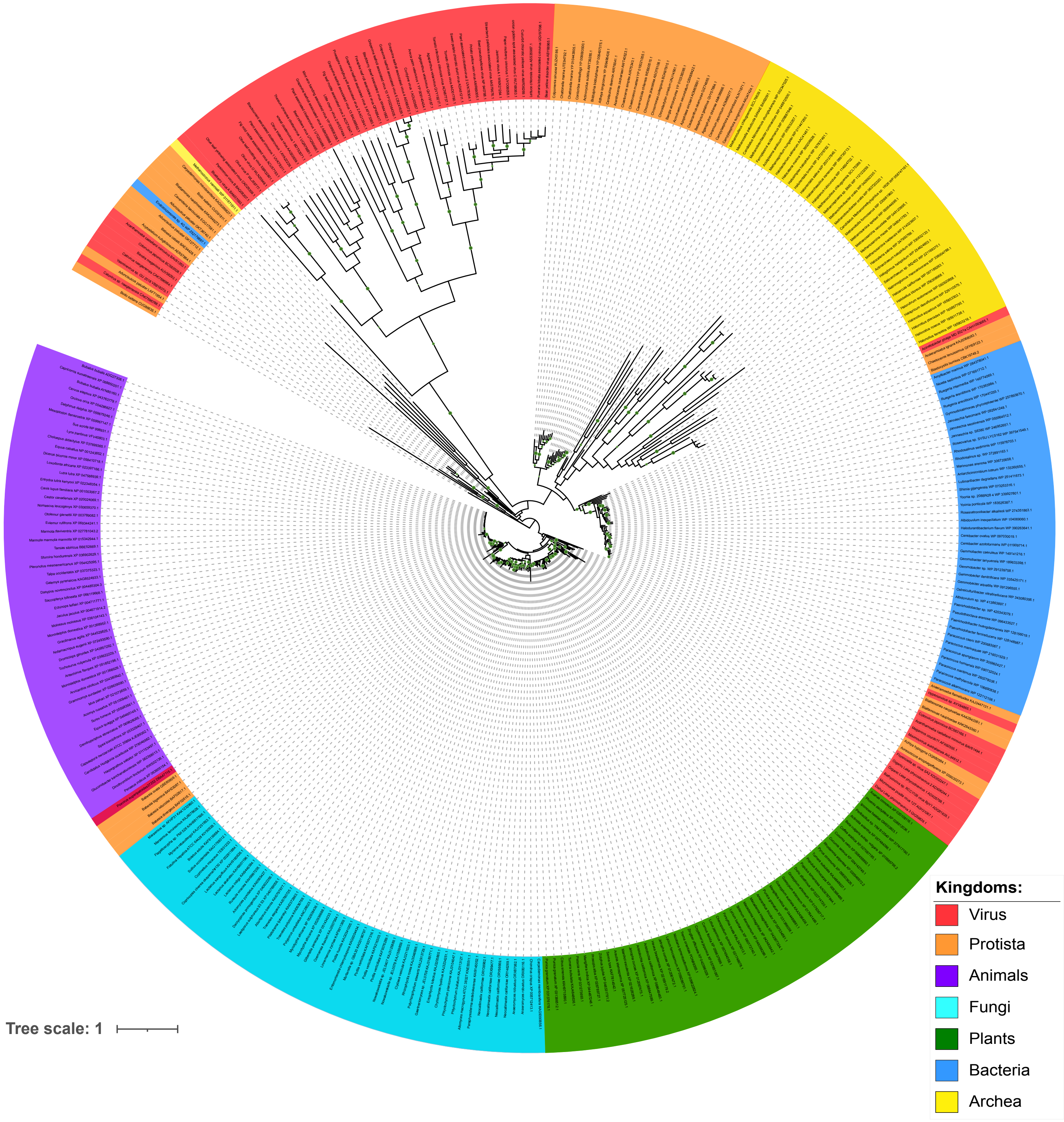

Figure S6. Phylogenetic relationships between viral and cellular HSP70s. Tree includes sequences from viruses, plants, fungi, protists, animals, and archaea. It is constructed using RAxML with the PROTGAMMALG model. Bootstrap values > 70% (1000 replicates) are shown as dots in green.

Figure S7

A

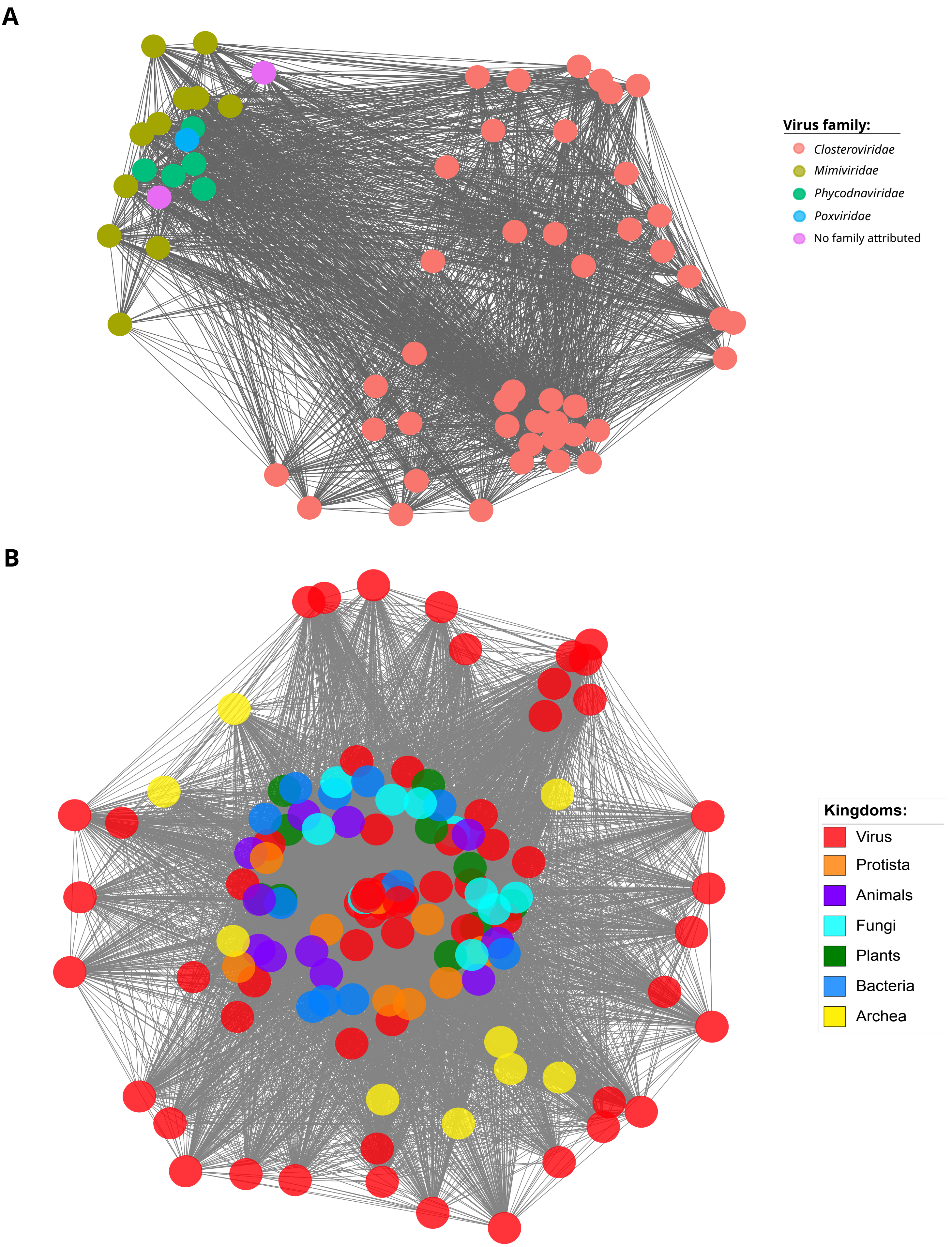

Figure S7. Sequence similarity network of viral and cellular HSP70 proteins generated using the EFI-EST tool. (A) Network of the viral HSP70s using a relaxed alignment score threshold of 10. All sequences form a single connected network, but the spatial separation of nodes still reflects two major subgroups, consistent with functional and evolutionary divergence. (C) Network including HSP70s from viruses and representative cellular organisms (10 species each from plants, animals, fungi, protists, bacteria, and archaea), constructed using the relaxed threshold (10). HSP70s from ssRNA viruses form clustered at the edges of the network, while those from dsDNA viruses integrate with cellular HSP70s, mainly from protists.
